# Perineural and Lymphovascular Invasion in Resected Pancreatic Ductal Adenocarcinoma: A High-Risk Subgroup That Could Benefit from Adjuvant Radiotherapy

**DOI:** 10.3390/jcm14217763

**Published:** 2025-11-01

**Authors:** Tufan Gümüş, Aykut Özkılıç, Recep Temel, Deniz Nart, Funda Yılmaz, Bülent Karabulut, Alper Uğuz

**Affiliations:** 1 Department of General Surgery, Ege University, Izmir 35100, Turkey; dr.tufan.gumus@gmail.com (T.G.); aykutozkilic.16@gmail.com (A.Ö.); doktorreceptemel@gmail.com (R.T.); 2 Department of Pathology, Ege University, Izmir 35100, Turkey; deniznart@yahoo.com (D.N.); funda.yilmaz@ege.edu.tr (F.Y.); 3 Department of Medical Oncology, Acıbadem Kent Hospital, Izmir 35630, Turkey; onkologbk@gmail.com

**Keywords:** pancreatic ductal adenocarcinoma, perineural invasion, lymphovascular invasion, adjuvant radiotherapy, recurrence, survival

## Abstract

**Background:** Perineural invasion (PNI) and lymphovascular invasion (LVI) are adverse prognostic factors in pancreatic ductal adenocarcinoma (PDAC). Their concurrence may warrant intensified adjuvant therapy. This study investigated the prognostic impact of concurrent PNI and LVI and the potential benefit of adjuvant radiotherapy. **Methods:** A retrospective analysis was conducted in patients who underwent pancreaticoduodenectomy for PDAC (2015–2023). Patients were grouped as PNI only, concurrent PNI and LVI, or neither. Clinicopathological features and survival outcomes were compared. In the concurrent PNI and LVI subgroup, the effect of adjuvant radiotherapy was analyzed according to margin status (R0 vs. R1). Disease-free survival (DFS) and overall survival (OS) were assessed using Kaplan–Meier and Cox models. **Results:** Eighty-eight patients were included. Recurrence occurred in 83.0%, with locoregional recurrence in 30.1%. Median DFS was 10.5 months and OS was 19.0 months; four patients (4.5%) survived ≥5 years. Concurrent PNI and LVI was observed in 55.7% and independently predicted reduced DFS (HR 3.23; *p* = 0.002) and OS (HR 2.34; *p* = 0.02), whereas adjuvant radiotherapy was associated with prolonged DFS (HR 0.48; *p* = 0.008). Among R0-resected patients with concurrent PNI and LVI, radiotherapy reduced locoregional recurrence (6.3% vs. 46.7%; *p* = 0.01) and improved median DFS (10.0 vs. 7.0 months; *p* = 0.04). **Conclusions:** Patients with concurrent PNI and LVI are a high-risk subgroup of PDAC. Adjuvant radiotherapy after R0 resection may improve DFS and reduce recurrence in these patients.

## 1. Introduction

The incidence of pancreatic cancer is estimated to be rising at a rate of 1.1% per year, with projections suggesting it will reach 18.6 cases per 100,000 individuals by 2050. Despite this increase in incidence, therapeutic advances have only led to modest improvements in outcomes. The five-year survival rate for pancreatic ductal adenocarcinoma (PDAC) remains at approximately 10%, with fewer than one in five patients eligible for surgical resection at diagnosis [1,2,3,4]. Therefore, accurate risk stratification based on histopathological and clinical features is essential for guiding decisions on adjuvant treatment and improving postoperative outcomes.

Perineural invasion (PNI) and lymphovascular invasion (LVI) are histopathological features that are often linked to a poor prognosis in PDAC. PNI, which is observed in 70–80% of cases, has been linked to reduced disease-free and overall survival, early recurrence, and increased local invasiveness [5,6,7]. Similarly, LVI has been associated with increased lymph node metastasis and distant dissemination, both of which negatively impact survival outcomes [8,9,10]. The presence of both PNI and LVI may indicate an aggressive tumour phenotype, although data on their combined prognostic impact remain limited [11].

The role of adjuvant radiotherapy in PDAC remains controversial. While some studies have demonstrated improved locoregional control in patients with high-risk pathological features, such as positive resection margins, nodal involvement or a large tumour size, other studies have shown limited, or no survival benefit compared with chemotherapy alone [12,13,14,15,16]. In particular, the impact of adjuvant radiotherapy in patients who have undergone R0 resection and present with both PNI and LVI remains unclear.

In this retrospective single-centre study, we aimed to investigate the prognostic relevance of PNI and LVI present in resected PDAC, and to evaluate whether adjuvant radiotherapy offers any benefit in terms of survival or recurrence in this high-risk subgroup.

## 2. Method

**Figure 1:**
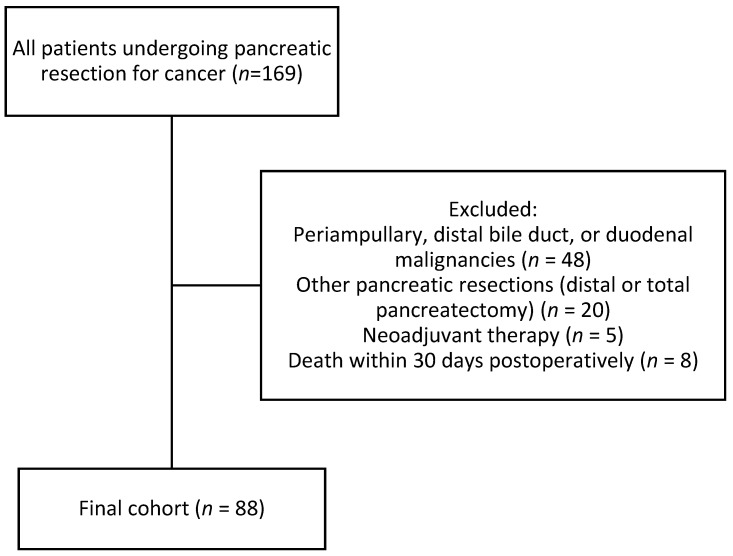
Flow diagram of patient selection.

We conducted a retrospective evaluation of patients who underwent pancreatic cancer surgery at our institution between January 2015 and July 2023. The study included patients who underwent pancreaticoduodenectomy and had a histologically confirmed diagnosis of pancreatic ductal adenocarcinoma (PDAC). Patients with periampullary, distal common bile duct, or duodenal malignancies (*n* = 48) were excluded, as were those who underwent other types of pancreatic resection, such as distal or total pancreatectomy (*n* = 20). Patients who received neoadjuvant therapy (*n* = 5) or who died within 30 days postoperatively due to surgical complications rather than tumour biology (*n* = 8) were also excluded (Figure 1).

Clinical data were collected and analysed, including age, sex, receipt of adjuvant chemotherapy and radiotherapy, recurrence status and location, histopathological features, disease-free survival (DFS), and overall survival (OS). DFS and OS were calculated from the date of histopathological diagnosis, with censoring at the last follow-up.

Recurrence patterns were categorized as locoregional, liver, or systemic, and were assessed over the entire follow-up period. Systemic recurrence was defined as the development of distant metastases outside the liver, including peritoneal, pulmonary, osseous, and cerebral sites, among others. Patients who developed sequential recurrences at different sites (e.g., locoregional recurrence followed by subsequent liver metastasis or peritoneal dissemination) were counted in each relevant category. Thus, the recurrence categories were overlapping rather than mutually exclusive. All recurrences were adjudicated based on radiological evidence and clinical documentation.

Pathology reports were retrospectively reviewed. Histopathological evaluation, routinely performed at our institution, included assessment of microscopic intratumorally lymphovascular invasion (LVI) and perineural invasion (PNI), tumour size, lymph node involvement, resection margin status, and tumour differentiation. LVI and PNI were assessed using haematoxylin and eosin (H&E) stained sections; when not evident on H&E, additional CD34 immunohistochemical staining was performed to confirm vascular invasion. Resection margin (R0/R1) status was defined according to the 0-mm rule, which represents the standard practice in our institution. All margins, including the retroperitoneal and superior mesenteric artery (SMA) surfaces, were inked and sliced perpendicularly, and this 0-mm definition was consistently applied throughout the study. Tumour size and nodal status were classified according to the 8th edition of the American Joint Committee on Cancer (AJCC) TNM staging system, and tumour differentiation was categorised as well, moderately, or poorly differentiated.

Adjuvant radiotherapy was delivered using intensity-modulated radiotherapy (IMRT) with 2-mm multileaf collimators and daily image guidance. A total dose of 50.4 Gy in 28 fractions was prescribed. The clinical target volume (CTV), which included the tumour bed and paraaortic/peripancreatic regions, and was expanded to generate the planning target volume (PTV). Dose constraints for adjacent organs at risk were applied according to institutional standards. Radiotherapy was typically initiated within 6–8 weeks after surgery. Concurrent chemotherapy consisted primarily of gemcitabine or gemcitabine-based combinations, and all patients received adjuvant chemotherapy. In our institution, decisions regarding postoperative radiotherapy were made in a multidisciplinary tumour board, and treatment was tailored to individual patient characteristics and pathological risk factors.

Patients were stratified into three groups according to invasion status: PNI-positive only, both PNI- and LVI-positive, and both negative. In the subgroup with concurrent PNI and LVI, further analyses were performed based on resection margin status (R0 vs. R1) and receipt of adjuvant radiotherapy. Among R0-resected patients in this subgroup, baseline characteristics were generally comparable between those who did and did not receive radiotherapy, with no significant differences in age, sex distribution, TNM stage, tumour differentiation, or chemotherapy regimens. Continuous variables were reported as mean ± standard deviation (SD) or median with interquartile range (IQR), depending on distribution. Categorical variables were summarised as frequencies and percentages. Group comparisons were conducted using the Chi-square test or Fisher’s exact test for categorical variables and Student’s *t*-test or Mann–Whitney U test for continuous variables, as appropriate.

The influence of tumour differentiation, T stage, N stage, resection margin status, PNI, and LVI on OS and DFS was assessed using Kaplan–Meier survival analysis, with differences between survival curves evaluated by the log-rank test. Variables identified as potentially prognostic in univariate analysis were further examined using multivariate Cox proportional hazards regression modelling. Statistical analyses were performed with IBM SPSS Statistics, version 25.0 (IBM Corp., Armonk, NY, USA), and a *p*-value < 0.05 was considered statistically significant.

## 3. Results

A total of 88 patients were included in the study. The median age was 65 years (IQR, 58.7–71.0), and 53 patients (60.2%) were male. The median follow-up was 19 months. Most patients (93.2%, *n* = 82) received adjuvant chemotherapy, and 36 patients (40.9%) underwent adjuvant radiotherapy. Recurrence occurred in 79 patients (83.0%), with liver metastases in 72.6%, systemic metastases in 74.0%, and locoregional recurrence in 30.1%. Overall mortality was 85.2% (*n* = 75), and long-term survival beyond five years was achieved in only four patients (4.5%). The median DFS was 10.5 months (IQR, 5.3–23.3), and the median OS was 19.0 months (IQR, 10.0–34.0).

Histopathological staging revealed T1 tumours in 12 patients (13.6%), T2 tumours in 45 (51.1%), and T3 tumours in 31 (35.2%). Nodal involvement was N0 in 24 patients (27.3%), N1 in 25 (28.4%), and N2 in 39 (44.3%). Tumours were well differentiated in 12 patients (13.6%), moderately differentiated in 66 (75.0%), and poorly differentiated in 10 (11.4%). An R0 resection was achieved in 64 patients (72.7%), whereas 24 (27.3%) had R1 margins. All patients with LVI also demonstrated PNI. PNI alone was observed in 25 patients (28.4%), both PNI and LVI were observed in 49 (55.7%), and neither feature was observed in 14 (15.9%).

Univariate analysis showed that patients with both PNI and LVI had significantly higher rates of poor tumour differentiation (18.4%) and R1 resection (36.7%) compared with the other groups (*p* = 0.03 and *p* = 0.04, respectively). Although recurrence and mortality rates were also higher in this subgroup, the differences did not reach statistical significance. Locoregional recurrence was most frequent in patients with concurrent PNI and LVI (34.7% vs. 12.0% and 14.3%; *p* = 0.06). Median DFS was significantly shorter in the concurrent PNI and LVI group (7.0 months) compared with 12.0 months in the PNI-only group and 36.0 months in patients without either feature (*p* < 0.001). Similarly, median OS was reduced in patients with both PNI and LVI (14.0 months) compared with 19.0 months in the PNI-only group and 43.0 months in those without either feature (*p* = 0.001) (Table 1).

Kaplan–Meier survival analysis confirmed these findings. Poorly differentiated tumours were associated with shorter DFS (5.0 months; 95% CI: 1.9–8.0) compared with well-differentiated tumours (14.0 months; 95% CI: 10.6–17.3). R1 resection was linked to shorter DFS (6.0 months vs. 11.0 months for R0; *p* = 0.07). Nodal stage was significantly associated with DFS: N0 patients had a median DFS of 16.0 months, N1 patients had a median DFS of 13.0 months, and N2 patients had a median DFS of 7.0 months (*p* = 0.003). The shortest DFS was observed in patients with both PNI and LVI (7.0 months) compared with 12.0 months in the PNI-only group and 36.0 months in those without either feature (*p* = 0.002). In multivariable Cox regression, concurrent PNI and LVI remained an independent predictor of reduced DFS (HR: 3.25; 95% CI: 1.53–6.88; *p* = 0.002). Conversely, receipt of adjuvant radiotherapy was independently associated with prolonged DFS (HR: 0.48; 95% CI: 0.28–0.82; *p* = 0.008). The overall Cox regression model for DFS was statistically significant (Omnibus test: χ^2^ = 31.9; df = 11; *p* = 0.001).

Kaplan–Meier analysis also confirmed a significant difference in overall survival (OS) according to nodal stage, with a median OS of 21.0 months (95% CI: 16.1–25.8) in N0 patients, 21.0 months (95% CI: 16.7–27.2) in N1, and 16.0 months (95% CI: 9.8–22.1) in N2 (*p* = 0.01). Patients without PNI or LVI demonstrated the longest OS at 43.0 months (95% CI: 30.4–55.5) compared with 22.0 months (95% CI: 16.2–27.7) in the PNI-only group and 14.0 months (95% CI: 9.4–18.5) in the concurrent PNI and LVI group (*p* = 0.005). In multivariable analysis, concurrent PNI and LVI remained the sole independent predictor significantly associated with reduced OS (HR: 2.34; 95% CI: 1.12–4.84; *p* = 0.02). The overall Cox regression model for OS was statistically significant (Omnibus test: χ^2^ = 19.7; df = 11; *p* = 0.049) (Table 2 and Table 3).

**Figure 2:**
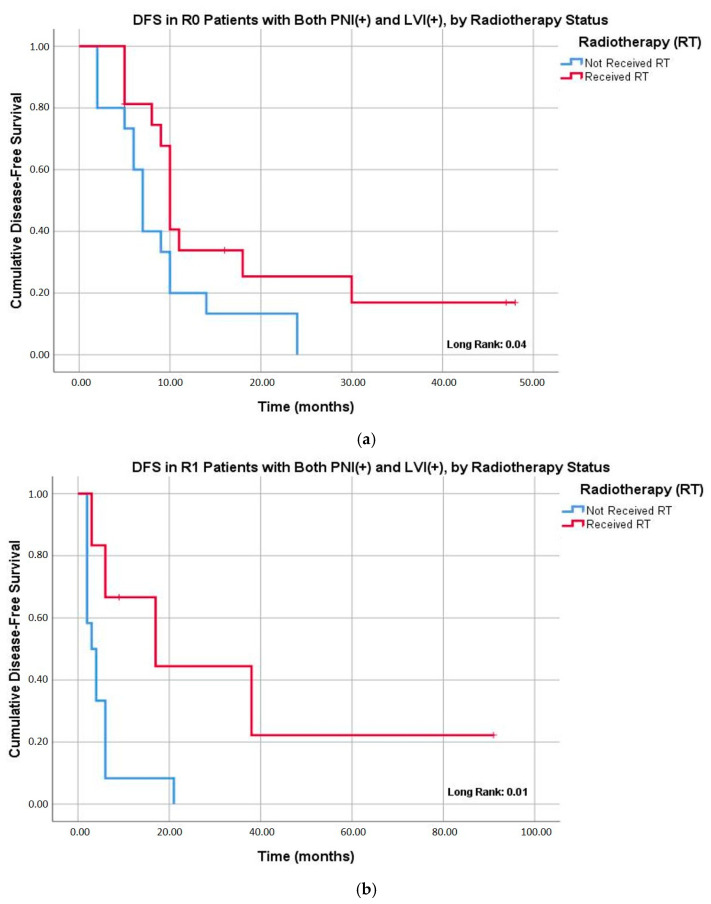
Kaplan–Meier analysis of disease-free survival (DFS) in patients with both the presence of PNI and LVI stratified by receipt of adjuvant radiotherapy. (**a**) The effect of adjuvant radiotherapy on DFS in patients undergoing R0 resection. The median DFS was 10.0 months (95% CI: 9.0–10.9) in the radiotherapy group compared to 7.0 months (95% CI: 5.7–8.2) in the non-radiotherapy group (*p* = 0.04). (**b**) The effect of adjuvant radiotherapy on DFS in patients undergoing R1 resection. The median DFS was 17.0 months (95% CI: not estimable–38.5) in the radiotherapy group compared to 3.0 months (95% CI: 0.7–5.2) in the non-radiotherapy group (*p* = 0.01).

Baseline clinicopathological characteristics of patients with concurrent PNI and LVI did not differ significantly according to receipt of adjuvant radiotherapy (Table S1). Subgroup analyses showed that among patients with both PNI and LVI who underwent R0 resection, adjuvant radiotherapy markedly reduced the risk of locoregional recurrence (6.3% vs. 46.7%; *p* = 0.01). A similar effect was observed for overall recurrence rates (75.0% vs. 100.0%; *p* = 0.03) (Table 4). Kaplan–Meier curves further demonstrated improved DFS with adjuvant radiotherapy in this subgroup: the median DFS was 10.0 months with radiotherapy versus 7.0 months without (*p* = 0.04). Notably, this effect was even more pronounced in R1 patients, where radiotherapy extended DFS to 17.0 months compared with 3.0 months in those without radiotherapy (*p* = 0.01) (Figure 2).

## 4. Discussion

To the best of our knowledge, this is the first study to specifically evaluate patients with concurrent PNI and LVI as a distinct subgroup. Previous studies have predominantly assessed PNI and LVI separately, consistently demonstrating their individual associations with poor prognosis. In our cohort, patients with both features had significantly shorter DFS and OS compared with those with only PNI or with neither feature, and concurrent PNI and LVI remained an independent predictor of recurrence and mortality in multivariable analysis. This novel perspective underscores the additive prognostic impact of PNI and LVI and highlights the importance of considering them jointly in future risk stratification models.

Prior studies have consistently reported that PNI is associated with early recurrence and worse survival, even after R0 resection, and that infiltration of retroperitoneal neural plexuses contributes to high local recurrence rates [17]. Existing studies have also shown that PNI is related to early recurrence and reduced OS and DFS [6,18]. Similarly, Crippa et al. reported that PNI independently predicted both local and systemic recurrence [7]. Schorn et al. demonstrated that PNI adversely affects prognosis even in patients with R0 resection. In addition, Schouten et al. highlighted the prognostic role of PNI in patients with R0 and N0 disease. Tanaka et al. found that the presence of PNI is associated with peritoneal dissemination [19,20,21]. Finally, a meta-analysis confirmed that neural invasion is an independent predictor of both DFS and OS [5].

Similarly, LVI has been associated with nodal metastasis and poor survival, although its prognostic strength has varied across studies. Şahin et al. reported that PNI had a stronger impact on survival than LVI, whereas Yamada et al. and Takahashi et al. suggested that microscopic venous and lymphovascular invasion were more strongly linked to adverse outcomes [8,9,10]. Epstein et al. demonstrated that LVI predicts lymph node metastasis and is an independent risk factor for reduced survival [22]. Similarly, Chen et al. further showed that patients with both PNI and LVI had the poorest long-term survival [11]. Our study extends these findings by evaluating patients with concurrent PNI and LVI as a distinct subgroup and demonstrating that their coexistence independently predicts an adverse prognosis with significantly reduced DFS and OS.

The role of adjuvant radiotherapy in pancreatic cancer remains debated. Hsu et al. reported that adjuvant chemoradiotherapy was associated with improved overall survival regardless of resection margin status [12]. In contrast, Van Laethem et al. found no significant differences in recurrence or survival between patients treated with adjuvant gemcitabine with or without radiotherapy [15]. A SEER-based study by Hazard et al. demonstrated that adjuvant radiotherapy improved survival in patients with T3, N1, or R1 disease, while Moaven et al. reported favourable outcomes in high-risk patients, particularly those with R1 resections, LVI, or nodal metastases [13,14]. Similarly, Parikh et al. observed recurrence and survival benefits in the R0 subgroup receiving chemoradiotherapy, although no significant advantage over chemotherapy alone was identified across the entire cohort [16]. More recently, a systematic review and clinical practice guideline concluded that adjuvant chemotherapy—preferably mFOLFIRINOX—represents the standard of care following PDAC resection, whereas there is insufficient evidence to support routine use of adjuvant radiotherapy or SBRT, except in selected high-risk subgroups or within clinical trials [23].

In our cohort, multivariable Cox regression analysis demonstrated that adjuvant radiotherapy was independently associated with improved disease-free survival (HR 0.48, 95% CI 0.28–0.82; *p* = 0.008), but did not confer a statistically significant benefit in overall survival (HR 0.67, 95% CI 0.40–1.13; *p* = 0.14). Adjuvant chemotherapy, on the other hand, was not identified as an independent prognostic factor. This finding is likely explained by the fact that 93.2% of patients in our study received adjuvant chemotherapy, which substantially reduced the ability to detect survival differences between treated and untreated groups.

In our institution, patients in this high-risk subgroup are routinely discussed in a multidisciplinary tumour board, where postoperative radiotherapy decisions are made on an individualized basis according to clinical and pathological features. In our subgroup analyses, patients with concurrent PNI and LVI who underwent R0 resection derived meaningful benefit from adjuvant radiotherapy, showing significantly lower rates of locoregional recurrence and improved disease-free survival (10 vs. 7 months). Despite margin-negative surgery, they remained at elevated risk of locoregional failure but appeared to respond favourably to radiotherapy.

These findings are particularly relevant because, to our knowledge, no previous study has specifically examined this subgroup of patients, and our results provide novel evidence that adjuvant radiotherapy may improve locoregional control in this setting. The absence of an overall survival benefit, despite improved local control, is likely attributable to the high incidence of systemic metastases, which are known to represent the predominant cause of mortality in PDAC. Furthermore, the relatively small sample size may have limited the statistical power to detect a survival difference, raising the possibility that a larger cohort could reveal a significant effect of adjuvant radiotherapy on overall survival.

Biologically, concurrent PNI and LVI likely reflect enhanced tumour dissemination through perineural and lymphatic pathways, especially into the retroperitoneum, thereby conferring a high risk of locoregional recurrence even after R0 resection. Experimental studies have shown that PNI in PDAC is an active process involving reciprocal tumour–nerve signalling via neurotrophic factors (e.g., NGF, GDNF, CXCL12), extracellular matrix remodelling, and Schwann cell activation, which together create a permissive niche for perineural invasion [24]. This biology may underlie the aggressive course observed in our cohort and provide a rationale for intensified adjuvant strategies, including radiotherapy, in this high-risk group.

In conclusion, while the adverse prognostic impact of PNI and LVI has been individually established, our study is among the first to show that their concurrent presence defines a particularly aggressive subgroup of PDAC with distinctly worse outcomes. Our findings suggest that adjuvant radiotherapy—even after R0 resection—could reduce locoregional recurrence and improve disease-free survival in these patients. Despite currently receiving standard adjuvant chemotherapy similar to other patients, we believe that this high-risk group may warrant more intensive adjuvant strategies. Moreover, if reliable preoperative predictors of PNI and LVI can be established, such patients, even if technically resectable, might be considered for neoadjuvant treatment to better address their aggressive disease biology. These results highlight the potential value of incorporating concurrent PNI and LVI into future risk stratification models and treatment guidelines to better tailor adjuvant therapy.

## 5. Limitations

This study has several limitations that should be acknowledged. Its retrospective, single-centre design may have introduced selection bias and inevitably limits the generalisability of the findings. The relatively small sample size, particularly in subgroup analyses, reduced the statistical power of some comparisons. Although radiotherapy was delivered according to a standard protocol, treatment adherence, potential interruptions, and toxicity profiles were not systematically assessed. Adjuvant chemotherapy regimens were heterogeneous, including gemcitabine, FOLFIRINOX, and combination therapies, which may have confounded the observed effects of radiotherapy. Finally, the non-randomized allocation of adjuvant radiotherapy introduces an inherent risk of selection bias; while baseline characteristics were broadly comparable, unmeasured variables such as performance status or comorbidities may still have influenced treatment decisions and outcomes.

## Figures and Tables

**Table 1:** Univariate analysis of patient characteristics based on the presence of perineural invasion (PNI) and lymphovascular invasion (LVI).

	Only PNI (+) (*n* = 25)	PNI and LVI (+) (*n* = 49)	PNI and LVI (−) (*n* = 14)	*p*-Value
Age (median, 95% CI)	69 [63.06–72.62]	64 [60.77–65.68]	70.0 [60.71–71.43]	0.17
Gender				
Male	14 (56.0%)	32 (65.3%)	7 (50.0%)	
Female	11 (44.0%)	17 (34.7%)	7 (50.0%)	0.51
Tumor differentiation				
Well	5 (20.0%)	3 (6.1%)	4 (28.6%)	
Moderate	19 (76.0%)	37 (75.5%)	10 (71.4%)	
Poor	1 (4.0%)	9 (18.4%)	0	**0.03**
T Stage				
T1	6 (24.0%)	4 (8.2%)	2 (14.3%)	
T2	14 (56.0%)	25 (51.0%)	6 (42.9%)	
T3	5 (20.0%)	20 (40.8%)	6 (42.9%)	0.23
N Stage				
N0	9 (36.0%)	10 (20.4%)	5 (35.7%)	
N1	9 (36.0%)	11 (22.4%)	5 (35.7%)	
N2	7 (28.0%)	28 (57.1%)	4 (28.6%)	0.11
R resection				
R0	20 (80.0%)	31 (63.3%)	13 (92.9%)	
R1	5 (20.0%)	18 (36.7%)	1 (7.1%)	**0.04**
Adjuvant CT	23 (92.0%)	46 (93.9%)	13 (92.9%)	0.95
CT regime				
GEM	2 (8.0%)	3 (6.1%)	1 (7.1%)	0.95
GEM + CAP	11 (44.0%)	19 (38.8%)	8 (57.1%)	0.47
FOLFIRINOX	5 (20.0%)	12 (24.5%)	2 (14.3%)	0.69
GEM + Platinum	4 (16.0%)	11 (22.4%)	1 (7.1%)	0.40
CAP	1 (4.0%)	1 (2.0%)	1 (7.1%)	0.63
Adjuvant radiotherapy	10 (40.0%)	22 (44.9%)	3 (21.4%)	0.28
Recurrence				
Yes	20 (80.0%)	43 (87.8%)	10 (71.4%)	0.32
Recurrence location				
Liver	13 (52.0%)	33 (67.3%)	7 (50.0%)	0.30
Locoregional	3 (12.0%)	17 (34.7%)	2 (14.3%)	0.06
Systemic	17 (68.0%)	28 (57.1%)	9 (64.3%)	0.64
Mortality	20 (80.0%)	44 (89.8%)	11 (78.6%)	0.39
DFS (median, 95% CI)	12.0 [11.41–26.67]	7.0 [7.85–16.89]	34.0 [21.52–50.19]	**<0.001**
OS (median, 95% CI)	19.0 [17.93–33.83]	14.0 [14.44–23.6]	39.0 [27.31–53.97]	**0.001**

DFS: Disease-Free Survival, OS: Overall Survival, PNI: Perineural invasion, LVI: Lymphovascular invasion, CT: Chemotherapy GEM: Gemcitabine, GEM+CAP: Gemcitabine + Capecitabine, CAP: Capecitabine, FOLFIRINOX: Folinic acid + Fluorouracil + Irinotecan + Oxaliplatin. Bold values indicate statistically significant results (*p* < 0.05).

**Table 2:** A comparison of median disease-free survival and overall survival according to clinicopathological factors, using Kaplan–Meier analysis.

	DFS (Months)			OS (Months)		
	Median	95% CI	*p*-Value	Median	95% CI	*p*-Value
Differentiation						
Well	14.0	10.6–17.3		23.0	NA–48.4	
Moderate	11.0	9.3–12.6		19.0	14.6–23.3	
Poor	5.0	1.9–8.0	0.10	7.0	NA–22.4	0.19
T Stage						
T1	43.0	9.1–76.8		24.0	NA–65.5	
T2	10.0	7.8–12.1		19.0	15.0–22.9	
T3	10.0	5.7–14.2	0.10	20.0	9.0–30.9	0.18
N Stage						
N0	16.0	5.8–26.1		21.0	16.1–25.8	
N1	13.0	8.1–17.8		22.0	16.7–27.2	
N2	7.0	3.6–10.3	**0.002**	16.0	9.8–22.1	**0.01**
Resection margin						
R0	11.0	8.7–13.2		19.0	13.3–24.6	
R1	6.0	3.6–8.3	0.07	15.0	6.1–23.8	0.11
PNI and LVI						
Only PNI present	12.0	10.7–13.2		22.0	16.2–27.7	
PNI and LVI present	7.0	4.7–9.2		14.0	9.4–18.5	
PNI and LVI absent	36.0	23.7–48.2	**0.001**	43.0	30.4–55.5	**0.005**

NA: Lower bound of 95% CI could not be estimated due to insufficient events or censoring pattern. PNI: Perineural invasion; LVI: Lymphovascular invasion. Bold values indicate statistically significant results (*p* < 0.05).

**Table 3:** Multivariable Cox regression analysis of factors associated with disease-free and overall survival.

		DFS (Months)			OS (Months)		
	*Ref.*	HR	95% CI	*p*-Value	HR	95% CI	*p*-Value
Differentiation	*well*						
Moderate		1.28	0.61–2.68	0.49	1.24	0.58–2.62	0.56
Poor		1.39	0.48–4.01	0.53	1.48	0.52–4.18	0.45
T Stage	*T1*						
T2		1.80	0.70–4.61	0.21	1.64	0.63–4.31	0.30
T3		1.24	0.46–3.35	0.66	1.16	0.43–3.14	0.76
N Stage	*N0*						
N1		0.80	0.39–1.67	0.56	0.70	0.33–1.49	0.35
N2		1.63	0.83–3.19	0.15	1.24	0.63–2.45	0.52
Resection margin	*R0*						
R1		1.33	0.73–2.42	0.35	1.27	0.71–1.52	0.41
PNI and LVI	*PNI and LVI (−)*						
Only PNI present		2.09	0.95–4.61	0.06	1.57	0.73–3.35	0.24
PNI and LVI present		3.25	1.53–6.88	**0.002**	2.34	1.12–4.84	**0.02**
Adjuvant CT	*No-CT*	0.88	0.22–2.69	0.83	1.14	0.35–3.65	0.82
Adjuvant RT	*No-RT*	0.48	0.28–0.82	**0.008**	0.67	0.40–1.13	0.14

PNI: Perineural invasion; LVI: Lymphovascular invasion; CT: Chemotherapy; RT: Radiotherapy. Bold values indicate statistically significant results (*p* < 0.05).

**Table 4:** The impact of adjuvant radiotherapy (RT) on local and overall recurrence rates in patients with the presence of both perineural and lymphovascular invasion.

(**A**). Locoregional Recurrence				
Resection Status	RT	Not Recurred	Recurrence	*p* value
R0	Yes	15 (93.8%)	1 (6.3%)	
	No	8 (53.3%)	7 (46.7%)	**0.01**
R1	Yes	3 (50%)	3 (50%)	
	No	6 (50%)	6 (50%)	1.00
(**B**). All Recurrence Sites				
Resection Status	RT	Not Recurred	Recurrence	*p* value
R0	Yes	4 (25%)	12 (75%)	
	No	0 (0%)	15 (100%)	**0.03**
R1	Yes	2 (33.3%)	4 (66.7%)	
	No	0 (0%)	12 (100%)	**0.03**

Bold values indicate statistically significant results (*p* < 0.05).

## Data Availability

The data presented in this study are available on reasonable request from the corresponding author. The data are not publicly available due to ethical restrictions.

## Supplementary Materials

The following supporting information can be downloaded at: https://www.mdpi.com/article/10.3390/jcm14217763/s1, Table S1: Baseline clinicopathological characteristics of patients with concurrent PNI and LVI according to receipt of adjuvant radiotherapy.
