# Supplementary material for: Perineural and Lymphovascular Invasion in Resected Pancreatic Ductal Adenocarcinoma: A High-Risk Subgroup That Could Benefit from Adjuvant Radiotherapy
Source: J Clin Med. 2025 Nov 1;14(21):7763. doi: 10.3390/jcm14217763 (PMC12608663; doi:10.3390/jcm14217763)
Supplement: Supplementary file 1 [file jcm-14-07763-s001.zip › jcm-3870264-supplementary.pdf]

Table S1. Baseline clinicopathological characteristics of patients with concurrent PNI and LVI according to receipt of adjuvant radiotherapy

	Radiotherapy (+)	Radiotherapy (-)	p-value
**Age (median, 95% CI)**	68.0 (60.1-69.9)	61.0 (58.4-66.1)	0.26
**Gender**			
Male	13 (61.9%)	8 (38.1%)	
Female	3 (30.0%)	7 (70.0%)	0.09
**Tumor differentiation**			
Well	0	3 (100%)	
Moderate	14 (58.3%)	10 (41.7%)	
Poor	2 (50.0%)	2 (50.0%)	0.59
**T Stage**			
T1	2 (66.7%)	1 (33.3%)	
T2	8 (50.0%)	8 (50.0%)	
T3	6 (50.0%)	6 (50.0%)	0.86
**N Stage**			
N0	5 (71.4%)	2 (28.6%)	
N1	4 (66.7%)	2 (33.3%)	
N2	7 (38.9%)	11 (61.1%)	0.24
**Adjuvant CT received**	16 (53.3%)	14 (64.7%)	0.48
**CT regime**			
GEM	3 (100%)	0	0.22
GEM + CAP	8 (57.1%)	6 (42.9%)	0.72
FOLFIRINOX	3 (42.9%)	4 (57.1%)	0.68
GEM + Platinum	2 (33.3%)	4 (66.7%)	0.39

CT: Chemotherapy GEM: Gemcitabin, GEM+CAP: Gemcitabin + Capecitabine, CAP: Capecitabine
